# Strengths and opportunities to clinical trial enrollment among BIPOC, rural dwelling patients in the northwest United States: a retrospective study

**DOI:** 10.3389/fphar.2024.1309072

**Published:** 2024-01-25

**Authors:** Jamie M. Nelson, Elizabeth Johnson, Becky Kiesow, Bernadette McCrory, Jiahui Ma

**Affiliations:** ^1^ Billings Clinic, Collaborative Science and Innovation Department, Billings, MT, United States; ^2^ Mark and Robyn Jones College of Nursing, Montana State University, Bozeman, MT, United States; ^3^ Biomedical Innovation for Research and Development Hub, Montana State University, Bozeman, MT, United States; ^4^ Billings Clinic, Diabetes Research, Billings, MT, United States

**Keywords:** clinical trial enrollment, access barriers, rural, frontier, decentralized trial

## Abstract

**Introduction:** Clinical trials investigating the safety and efficacy of experimental drugs and devices are the cornerstone of medicinal advancement. Enrolling sufficient participants in these trials is vital to ensure adequate statistical power and generalizability. Clinical trial participation is particularly low among certain populations, including medically underserved communities (i.e., rural areas) and Black, Indigenous, and People of Color (BIPOC).

**Methods:** A retrospective study design was used to understand patient outcomes and access/barriers to clinical trial participation in the rural northwest United States. A quantitatively focused retrospective chart review was conducted for adult participants enrolled in at least one clinical trial in a single northwest health system between 1999 and 2022. Descriptive and inferential statistical analyses were performed to assess trial outcomes at a significance level 0.05.

**Results:** The retrospective chart review yielded 833 clinical trial records with 753 individual enrolled participants. The all-cause relative frequency of death at last known follow-up amongst clinical trial participants was 8.90% (n = 67). Based on logistic regression, the death was significantly associated with the participants’ age at initial trial screening (*β* = 0.09, *p*-value <0.001), those that resided in non-metro areas (*β* = −0.86, *p*-value = 0.045), and those that lived in Northeastern Montana (*β* = 1.27, *p*-value = 0.025). Additionally, death at last known follow-up was significantly associated with enrollment in 2021–2022 (*β* = −1.52, *p*-value <0.001), enrolled in more than one study (*β* = 0.84, *p*-value = 0.023), in internationally sponsored trials (*β* = −2.08, *p*-value <0.001), in Phase I (*β* = 5.34, *p*-value <0.001), in Phase II trials (*β* = 1.37, *p*-value = 0.013), diabetes as a primary trial target (*β* = −2.04, *p*-value = 0.003).

**Conclusion:** As decentralized trial design and remote or virtual elements of traditional trials become normative, representation of rural and frontier populations is imperative to support the generalizability of trial data encouraged by the FDA.

## 1 Introduction

Clinical trials (CTs), the cornerstone of medicinal advancement, offer patients the opportunity to receive state-of-the-art treatments and access to potentially effective options before they are approved for routine use (Krzyzanowska et al., 2011). Emerging technologies administered as part of CTs may improve health outcomes (i.e., cancer control) and improve quality of life or shorten treatment times, thus change the overall burden of a life-threatening illness. Over the past decade, the number of drugs developed for gynecological and breast cancers based on CTs has increased (Workman, 2003; Doisneau-Sixou and Harbeck, 2014; Beaver et al., 2019). Enrolling sufficient participants in these trials is vital to ensure adequate statistical power and generalizability. Yet, participation in CTs has remained low for more than 20 years, particularly among community sites (4%) when compared to National Cancer Institute (NCI) designated sites (19%) (Unger and Fleury, 2021). In addition to the practice site, health status, race and ethnicity, rurality, and socioeconomic status influence clinical trial enrollment (Caston et al., 2022a; Caston et al., 2022b).

Clinical trials should be equally accessible to all populations for many reasons, including access to novel treatments that may not otherwise be available. Specific populations are often underrepresented, including racial and ethnic minorities, uninsured, socioeconomically disadvantaged, elderly, and rural populations. This underrepresentation limits the ability to generalize trial results to diverse patient populations. Lack of CT representation is well documented for patients who are Black, Indigenous, People of Color (BIPOC), rural residents, or patients living in disadvantaged areas (Caston et al., 2022a; Caston et al., 2022b). Lack of CT representation is particularly low among indigenous populations; only 1% of participating individuals are American Indian/Alaska Native (AI/AN) - a disproportionately low level (Mainous et al., 2023). Patients in rural areas have decreased participation in clinical trials, with about one in three clinical trial participants are rural residents compared to one in five in the general population (Unger et al., 2018; Bharucha et al., 2021)). Multilevel barriers to clinical trial participation disproportionately affect certain groups that are prevalent in older adults and rural residents, resulting in underrepresentation in clinical trials (Hamel et al., 2016). Economic, social, cultural, and medical barriers to CT representation have been suggested, including unequal access to the healthcare system, mistrust of clinical research, poor past experiences with the healthcare system, lack of insurance, lack of transportation, and vast geographical distances to seek healthcare (Iglehart, 2018; Wercholuk et al., 2022).

Such discrepancies in CT participation opportunities may exacerbate known cancer-related health disparities among underserved populations. Differences in cancer risk and biology among underserved populations may also contribute to outcome disparities, especially if these groups are underrepresented in clinical trials, as the impact of newer therapies could be inadequately studied in these populations (Johnson et al., 2014; Geana et al., 2017; Batai et al., 2018; Rayford et al., 2021). For example, recent data from the Carolina Breast Cancer Study suggests that Black women more often have higher-risk, harder-to-treat breast cancer than women of other racial groups (Troester et al., 2018). While clinical trial participation is associated with decreased mortality (Unger et al., 2014; Unger et al., 2018), it should be noted that high area-level socioeconomic deprivation has been found to result in persistent disparities even with clinical trial enrollment (Unger et al., 2021). A SWOG Cancer Research Network report demonstrates that access to clinical trials narrows the gap in cancer care disparity among patients in urban and rural communities (Seidler et al., 2014; Perni et al., 2021).

In recognition of these issues and the challenges faced by the BIPOC, rural, and underserved individuals it serves, our large healthcare organization in the rural Northwest undertook a comprehensive effort to explore the strengths and opportunities of clinical trials specific to our region. Within our healthcare area, patients travel considerable distances (e.g., 500 miles) across the vast geographic expanse of Montana, Wyoming, and North Dakota to attend study visits. Unique structural and cultural factors further impede access to care within the region including avoidance of care, fear of anonymity, perceived lack of confidentiality due to small community, and mistrust of unfamiliar staffing (Burch, 2022). Diversity exists due to the rurality of the patient population and the limited number of specialists throughout the region. Due to the complexity of CTs, challenges occur in implementing virtual and regional models based on the availability and lack of trained staff in rural communities. Virtual visits can be implemented; however, limitations exist without adequately trained staff to conduct study-specific procedures. Yet, there remains a lack of literature exploring such limitations and opportunities for clinical trial participation among diverse populations. Therefore, the aims of this retrospective chart review were two-fold: 1) explore the strengths and opportunities of clinical trial participation among BIPOC, rural, and underserved individuals, and 2) objectively understand enrollment, utilization, and outcomes of clinical trial participants in the rural Northwest.

## 2 Materials and methods

### 2.1 Study design

This retrospective chart review (RCR) explored all known clinical trial participants who were provided care between 1999 and 2022 at a large healthcare organization in the rural Northwest. The quantitative data described participant attributes, including demographics, clinical trial study details, insurance information, and hospital discharge information.

#### 2.1.1 Data management

Clinicians and research staff employed at the large healthcare organization were responsible for all data collection and management aspects throughout the study period and subsequent dissemination. Investigators complied with data stewardship and other applicable standards for data collection, data entry and management, data analysis, and dissemination activities.

#### 2.1.2 Data collection

Given that this was an RCR, the request for waiver of informed consent for medical record review was sought and approved by the Montana State University Institutional Review Board (Protocol # 2023–604) and the hospital organization’s Privacy & Exemption Committee on 19 April 2023. After approval, a list of all the names and medical record numbers of adult patients (age 18 and over) with a record of clinical trial participation in calendar year 1999 (starting 01 January 1999) through calendar year 2022 (ending 31 December 2022) were collected and managed by healthcare research staff and clinicians. Data were then de-identified and securely transferred for statistical analyses. All consecutive patient charts were then retrospectively reviewed from a large healthcare system in the rural Northwest. This rural healthcare system has 40 affiliated facilities and a primary service area that spans Montana, northern Wyoming, and part of eastern North Dakota. The RCR identified all patients previously or currently enrolled in any clinical trial at any of the 40 affiliated facilities.

#### 2.1.3 Screening and inclusion criteria

**FIGURE 1:**
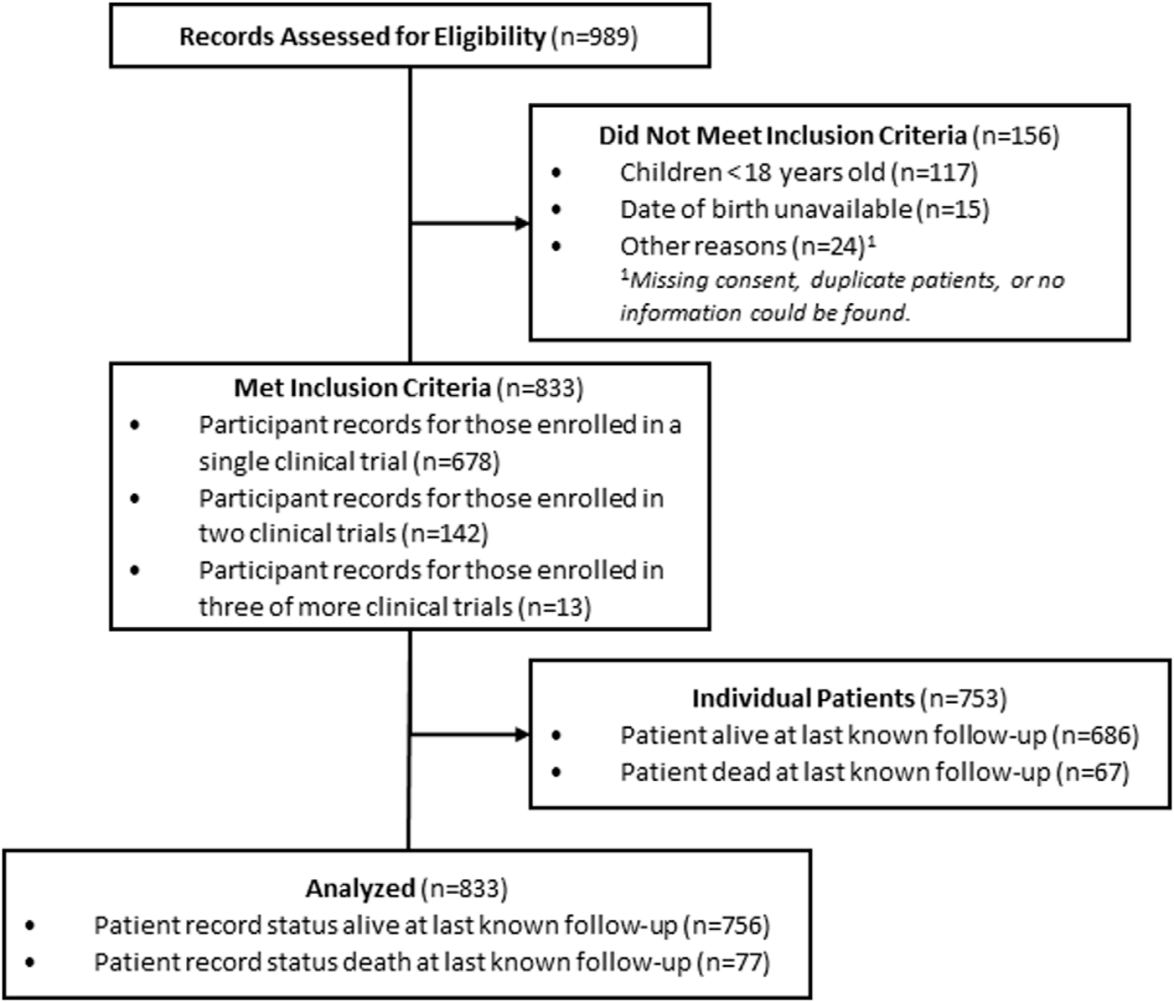
RCR chart screening, inclusion and analysis.

The healthcare system’s electronic and hardcopy medical records were reviewed to identify any patients enrolled in a clinical trial from 1999 to 2022. A total of 989 participant records were extracted from the electronic database and physical charts (Figure 1). Patients were included in this RCR if they were provided care at one of the 40 affiliated healthcare system sites; enrolled in at least one clinical trial between 1999 and 2022; and were adults aged ≥18 years at trial enrollment. Patients were excluded if not enrolled in a clinical trial between 1999 and 2022; children less than 18 years old; date of birth was missing/unavailable; or consent form(s) were missing/unavailable from electronic files. The final dataset encompassed 833 records, which included 753 individual patients (75 patients participated in more than one trial during the study period).

#### 2.1.4 Data attributes

The extracted dataset comprised a total of 20 key attributes. These attributes included participant demographic backgrounds, clinical trial study details, insurance information, and hospital discharge information (Table 1). Due to the limited number of participants enrolled in more than one study, the number of studies enrolled for each participant was summarized into two levels (one study vs. more than one study). To balance the study distribution across categories, the ten Rural-Urban Commuting Area (RUCA) Codes were recategorized into metropolitan (metro) areas and nonmetropolitan (nonmetro) areas. Both primary and secondary insurance types included commercial (e.g., BlueCross BlueShield), government (i.e., Medicaid, Medicare, Tricare, Veterans Affairs, and Indian Health Service), and other insurance types (i.e., no insurance, charity care, self-pay). Due to limited information (e.g., unreported), secondary insurance types were excluded from inferential analyses. However, a composite variable for known primary and secondary insurance types was included to understand access/barriers to advanced care, such as clinical trials. This composite insurance variable was summarized into three levels (i.e., no insurance, single insurance, two or more insurances). Study status, last encounter type, and days to a deceased endpoint were excluded from inferential analysis because of class imbalance (i.e., skewed distributions) for deceased participants. Lastly, the trial enrollment year and disease site were aggregated across levels based on expert input (EJ and JB) for inferential analyses.

**TABLE 1:** Extracted dataset attributes.

Attributes (total attributes k = 20)	Descriptive analysis	Inferential analysis
Age at screening	Continuous, years	Continuous, years
Number of studies enrolled	Dichotomous, 2 levels	Dichotomous, 2 levels: One study | More than one study
Gender	Dichotomous, 2 levels	Dichotomous, 2 levels: Female | Male
Marital status	Categorical, 3 levels	Categorical, 3 levels: Married & Live partner | Single | Divorced, Separated & Widowed
Race	Categorical, 3 levels	Categorical, 3 levels: White | American Indian or Alaska Native | Other
RUCA^a^	Dichotomous, 2 levels	Dichotomous, 2 levels: Metro Area | Nonmetro Area
RUCC^b^	Dichotomous, 2 levels	Dichotomous, 2 levels: Metro Area | Nonmetro Area
MT area code	Categorical, 4 levels	Categorical, 4 levels
		Northeastern | Eastern | North Central, South Central & Western | Outside MT
International study	Dichotomous, 2 levels	Dichotomous, 2 levels: Domestic | International
Trial sponsor type	Categorical, 4 levels	Categorical, 4 levels: Academic Institute/Healthcare Organization | Biotech/Device |
		Consortium/Network/Foundation | Pharmaceutical/Biopharmaceutical
Primary sponsor type	Categorical, 3 levels	Categorical, 3 levels: Commercial | Government | Other
Secondary insurance type	Categorical, 3 levels	-
Insurance status	Categorical, 3 levels	Categorical, 3 levels: Single Insurance | Two Insurances | No insurance
Study enrolled year	Categorical, 5 levels	Categorical, 2 levels: 2020 and prior | 2021–2022
Study status	Categorical, 6 levels	-
Study phases	Categorical, 6 levels	Categorical, 6 levels: Observational | Pilot | Phase I | Phase II | Phase III | Phase IV
Disease site	Categorical, 7 levels	Categorical, 3 levels: Cancer, Breast & GYN | Diabetes | Other
Deceased status	Dichotomous, 2 levels	Dichotomous, 2 levels: Deceased | Not deceased
Last Encounter type	Categorical, 4 levels	-
Days to deceased	Continuous, days	-

^a^ Metro area: RUCA, 1-6, Nonmetro area: RUCA, 7–10.

^b^ Metro area: RUCC, 1-3, Nonmetro area: RUCC, 4–9.

#### 2.1.5 Statistical analyses

Both descriptive and inferential analyses were completed using the R programming language (Version R-4.3.0) (R Core Team, 2013) with Tidyverse packages (Wickham et al., 2019). A descriptive analysis was conducted for all 20 attributes. Means and standard deviations were calculated for continuous attributes. Frequency and relative frequency were summarized for dichotomous and categorical attributes. Inferential analysis includes association and logistic regression analysis for 16 attributes (Table 1). Analysis of variance and Chi-square analyses were used to determine relationships amongst and between features. The logistic model used death at last known follow-up (yes, no) as the dependent variable and selected predictors (i.e., screening via backward selection method) analysis as independent variables (Borboudakis and Tsamardinos, 2019). Two-sided significance level was set at 0.05 for all inferential analyses.

## 3 Results

### 3.1 Patient characteristics

Of the 833 clinical trial participant records, 753 adult patients (75 participated in more than one trial during the study period) were included in this retrospective chart review (RCR). As detailed in Table 2, participants were, on average, 57.5 (standard deviation 15.0) years old at first clinical trial screening, most were female (62.2%), White (92.2%), and married/domestic partnership (63.6%). Patients’ primary health insurance included a nearly equivalent frequency of commercial (46.8%) and government (49.4%) insurance types. Over 33% of participants had two or more insurance types. A larger than expected proportion of 3.6% of older adults (≥80 years old) were enrolled in clinical trials.

**TABLE 2:** Patient demographics at first trial screening by status at last known follow-up.

	All patients (n = 753)	Alive (n = 686)	Dead (n = 67)
Age^a^	57.5 (15.1)	56.4 (15.0)	68.1 (11.4)
Age Interval^b^			
18–44	167 (22.2)	164 (23.9)	3 (4.5)
45–64	308 (40.9)	285 (41.5)	23 (34.3)
65–69	111 (14.7)	103 (15.0)	8 (11.9)
70–74	96 (12.8)	84 (12.2)	12 (17.9)
75–79	44 (5.8)	34 (5.0)	10 (14.9)
≥80	27 (3.6)	16 (2.3)	11 (16.4)
Female^b^	468 (62.2)	428 (62.4)	40 (59.7)
Marital Status^b, c^			
Married or Domestic Partnership^d^	479 (63.6)	438 (63.8)	41 (61.2)
Single	161 (21.4)	149 (21.7)	12 (17.9)
Divorced, Separated & Widowed	108 (14.3)	94 (13.7)	14 (20.9)
Race^b, c^			
White	694 (92.2)	632 (92.1)	62 (92.5)
American Indian or Alaska Native	34 (4.5)	30 (4.4)	4 (6.0)
Other^e^	22 (2.9)	21 (3.1)	1 (1.5)
RUCA Code^b, f^			
Metropolitan Counties	517 (68.7)	471 (68.7)	46 (68.7)
Nonmetropolitan Counties	236 (31.3)	215 (31.3)	21 (31.3)
Montana Region^b^			
North Eastern	73 (9.7)	64 (9.3)	9 (13.4)
Eastern	535 (71.1)	489 (71.3)	46 (68.7)
North Central	9 (1.2)	9 (1.3)	-
South Central	49 (6.5)	43 (6.3)	6 (9.0)
Western	9 (1.2)	8 (1.2)	1 (1.5)
Outside Montana^g^	78 (10.3)	73 (10.3)	5 (7.5)
Primary Insurance Type^b^			
Commercial	352 (46.8)	334 (48.7)	18 (26.9)
Government Insurance	372 (49.4)	327 (47.7)	45 (67.1)
Other	29 (3.8)	25 (3.6)	4 (6.0)
Insurance Status^b^			
Single Insurance	471 (62.6)	442 (64.4)	29 (43.3)
Two Insurances	253 (33.6)	219 (31.9)	34 (50.7)
No Insurance	29 (3.8)	25 (3.6)	4 (6.0)
Enrolled in more than one trial^b^	75 (10.0)	67 (9.8)	8 (11.9)

^a^ Mean (Standard Deviation).

^b^ Frequency (Relative Frequency).

^c^ Missing marital status (n* = 5) and Race (n* = 3).

^d^ Married (62.2%) and Domestic partnerships (1.46%).

^e^ Other races included Asian (1.20%), Black/African American (1.20%), Native Hawaiian/Pacific Islander (0.27%), multiple races/not reported (1.20%).

^f^ Defined using the rural-urban commuting area codes. RUCA, is a classification system used to categorize geographic areas based on their level of urbanization and commuting patterns.

^g^ Wyoming (8.76%), Minnesota (0.40%), South and North Dakota (0.40%), and other states (0.80%).

### 3.2 Clinical trial characteristics

There were 833 clinical trial participants from 1999 to 2022, supported by 753 individual patients (i.e., 75 patients were enrolled participants in more than one study) (Table 3). Most trials began in 2021 (209, 25.1%), with only a few trials per year from 1999 to 2018 (178, 21.4%). Domestic (US-based) clinical trials were most prevalent (601, 72.1%), and most trials were sponsored by biotechnology/medical device companies (292, 35.1%). While all study phases (pilot, phases I-IV, and observational) were conducted, a vast majority were observational trials (448, 53.8%). Trials primarily focused on the treatment of diabetes (34.1%), breast or gynecological cancer (20.6%), and other forms of cancer (19.6%). Due to specialty availability, very few Biobank/Repository (4.8%) and lung-related (3.0%) trials were conducted.

**TABLE 3:** Clinical trial characteristics.

	Trial participation (n = 833)	End of trial status: Alive (n = 756)	End of trial status: Dead (n = 77)
Trial enrollment year^a^			
2018 and prior	178 (21.4)	160 (21.2)	18 (23.4)
2019	150 (18.0)	126 (16.7)	24 (31.2)
2020	131 (15.7)	109 (14.4)	21 (28.6)
2021	209 (25.1)	198 (26.2)	11 (14.3)
2022	165 (19.8)	163 (21.6)	2 (2.6)
Domestic vs. International Trials^a^			
International Trials	232 (27.9)	216 (28.6)	16 (20.8)
Domestic Trials	601 (72.1)	540 (71.4)	61 (79.2)
Trial Sponsor Type^a^			
Academic Institute/Health Organization	178 (21.4)	169 (22.4)	9 (11.7)
Biotechnology/Device Company	292 (35.1)	277 (36.6)	15 (19.5)
Consortium/Network/Foundation	270 (32.4)	231 (30.6)	39 (50.6)
Pharmaceutical/Biopharmaceutical	93 (11.2)	79 (10.4)	14 (18.2)
Study Phases^a^			
Pilot	52 (6.2)	51 (6.7)	1 (1.3)
Phase I	10 (1.2)	3 (0.4)	7 (9.1)
Phase II	35 (4.2)	26 (3.4)	9 (11.7)
Phase III	169 (20.3)	158 (20.9)	11 (14.3)
Phase IV	119 (14.3)	104 (13.8)	15 (19.5)
Observational	448 (53.8)	414 (54.8)	34 (44.2)
Disease/Disorder Target^a^ ^,^ ^b^			
Biobank/Biospecimen Repositories	40 (4.8)	35 (4.6)	5 (6.5)
Breast/Gynecological Cancers	172 (20.6)	149 (19.7)	23 (29.9)
Cancer	163 (19.6)	135 (17.9)	28 (36.4)
Cardiovascular	50 (6.0)	38 (5.0)	12 (15.6)
Diabetes	284 (34.1)	277 (36.6)	7 (9.1)
Lung-related	25 (3.0)	24 (3.2)	1 (1.3)
Neurological-related	99 (11.9)	98 (13.0)	1 (1.3)
Trial Participant Enrollment Status^a^ ^,^ ^c^			
Complete	410 (49.2)	410 (54.2)	N/A
Deceased	77 (9.2)	-	77 (100.0)
Early Termination^d^	39 (4.7)	39 (5.2)	N/A
Trial Monitoring^e^	161 (19.3)	161 (21.3)	N/A
Randomized^f^	80 (9.6)	80 (10.6)	N/A
Screen Fail^g^	66 (7.9)	66 (8.7)	N/A

^a^ Frequency (Relative Frequency).

^b^ Biobank/Biospecimen Repositories (Biorepository, Caris Biorepository, Diabetes Related Antibodies, and Polycythemia Vera), Breast/Gynecological Cancers (Breast, Breast Pre-Biopsy Blood Collection, GYN, and Ovarian), Cancer (Circulating Tumor Cells, Multiple Myeloma, Cancer Health Disparities, Tumors, Cancer Central Nervous System, Colon Cancer, GI, glioblastoma; GU, Head & Neck Cancer, Leukemia, Lymphoma, Melanoma, Prostate Cancer, and Renal Cell), Cardiovascular (Endotak Reliance, Heart Failure, and Watchman Device–Afib), Diabetes (T1DM, T2DM, and Diabetes), Lung-related (Cystic Fibrosis, Lung, and Lung Cancer), Neurological-related (MS, and Pain).

^c^ NR: Not reportable due to sample size <5; N/A: Not Applicable.

^d^ Patients who either revoked consent or had to stop trial due to other circumstances (serious adverse events, moving out of area, etc.).

^e^ Patients complete with active treatment but were monitored for recurrence and death. This status is typically only used for cancer trials.

^f^ Patient signed consent, and was actively receiving treatment.

^g^ Patient signed consent, however, did not receive study drug. Usually excluded from study based on disease severity and/or specific inclusion/exclusion criteria of the clinical trial.

### 3.3 Trial participation by rurality

**FIGURE 2:**
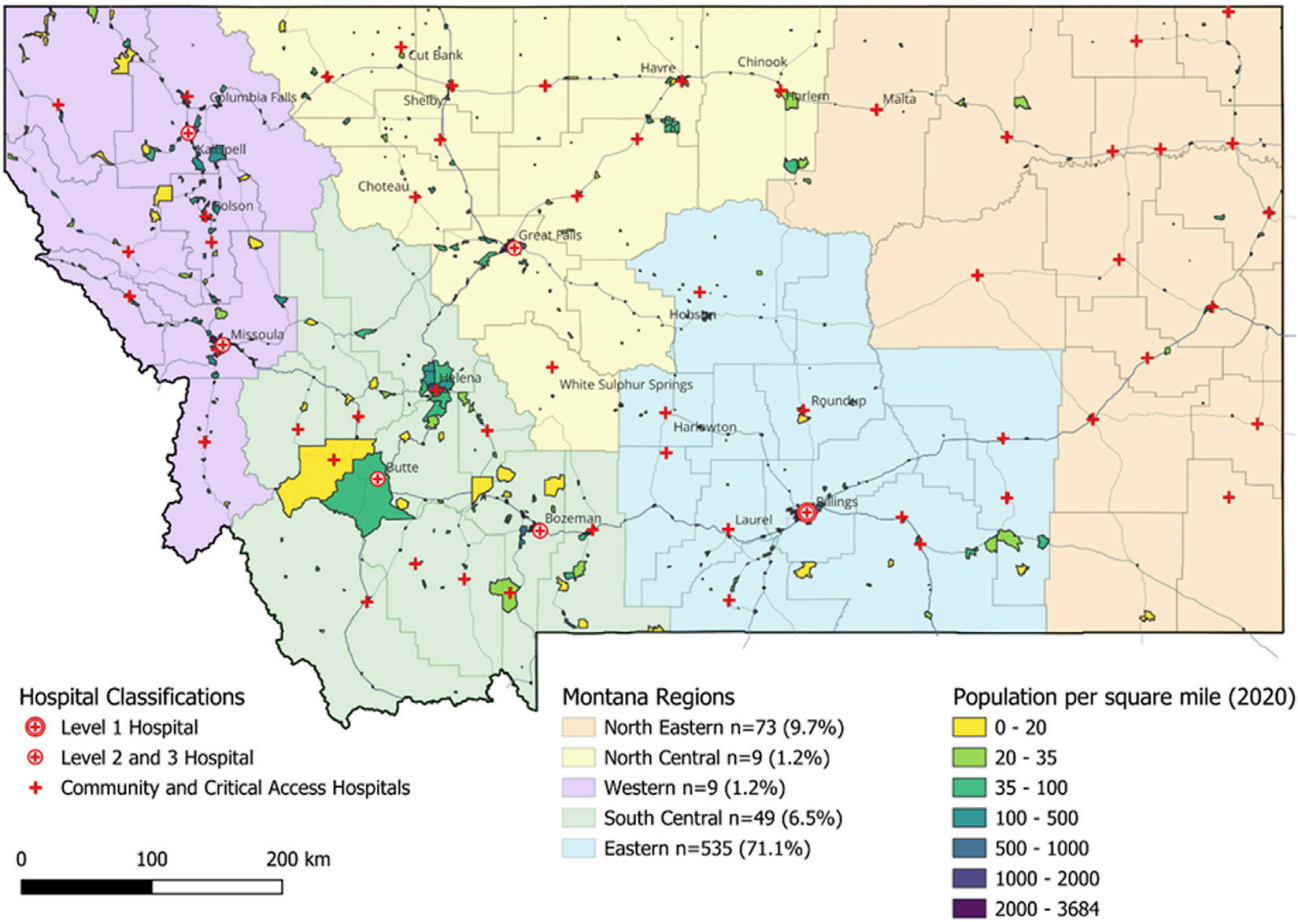
Hospital locations, population and trial participants across Montana Regions. 56 community and critical access hospitals. 6 level 2 and 3 hospitals. 1 level 1 hospital. Montana has three metro core areas: Billings, Missoula and Great Falls. And it has four micro core areas: Kalispell, Bozeman, Helena and Butte.

As of 2023, this large healthcare organization in the rural Northwest is the only Level I Trauma Center serving the states of Montana and Wyoming across 244,854 square miles (about the area of Texas), which are primarily designated as rural areas (i.e., 27.5% RUCA Code 10: primary flow to a tract outside of an urban area or urban center) (U.S. Department of Agriculture, 2019) (Figure 2). Patients from metropolitan tracts (i.e., RUCA codes 1 and 2) accounted for most trial participants (63%) (Table 4), yet nearly 22% of study participants lived in non-metropolitan areas (RUCA codes 4–8). Only 15% of participants lived in the most rural, isolated areas (RUCA 10). Across the state of Montana, most participants resided in Eastern Montana (71.9%), coinciding with the rural hospital’s main location. In conjunction with the hospital’s service area, the next greatest regional enrollment came from northern Wyoming (10.3%).

**TABLE 4:** Clinical trial participation comparison based on Montana RUCA Codes.

Primary RUCA Code^a^	Metropolitan or Non-Metropolitan^b^	MT RUCA tracts (n = 271)^c^	All patients (n = 753)^d^	Participation difference^e^ (%)
1	Metropolitan area core	61 (22.51%)	358 (47.54%)	+25.03
2	Metro area high commuting	23 (8.49%)	118 (15.67%)	+7.18
4 & 5	Non-metro micropolitan areas	51 (18.82%)	41 (5.44%)	−13.38
7	Non-metro, small-town core	39 (14.39%)	111 (14.74%)	+0.35
8	Non-metro, small-town high commuting	11 (4.06%)	13 (1.73%)	−2.33
10	Non-metro, rural areas	86 (31.73%)	112 (14.87%)	−16.86

^a^ Defined using the rural-urban commuting area codes. RUCA, is a classification system used to categorize geographic areas based on their level of urbanization and commuting patterns (U.S., department of agriculture, 2019). There are no RUCA, Codes 3 and 9 in the State of Montana; RUCA, codes 4 and 5 combined due to low sample sizes.

^b^ Metro counties are according to the population size of the metro area—those in “large” areas have at least 1 million residents and those in “small” areas have fewer than 1 million residents. Nonmetro counties include all counties outside metro areas and are classified as micropolitan, small town or rural area (U.S., department of agriculture, 2013).

^c^ Frequency (relative frequency) by total number of FIPS, tracts in that state of Montana is 271 (U.S., census bureau, 2017).

^d^ Frequency (relative frequency) for Montana patients.

^e^ Computed by subtracting the relative frequency of tracts by RUCA, code from study participation (All Patients-RUCA, Tracts).

### 3.4 End of trial status

Across all clinical trial participants, 678 were enrolled in only one study, and 75 participants were enrolled in two or more clinical trials (Table 2). A total of 67 participants were reported as deceased as the final clinical or follow-up endpoint for an all-cause relative frequency of death of 8.90%. Deceased participants were, on average, 10 years older than surviving patients. Over 22% of enrolled participants were aged 70 years and older. Half (49.4%) of deaths occurred in those aged 70 years and older. The frequency of death was marginally higher amongst females (59.7%) and those divorced/separated/widowed (20.9%). Deaths were higher in metropolitan tracts (67.5%) compared to non-metro tracts (32.5%).

Most deaths occurred amongst participants enrolled in one clinical trial (Table 5). Since only one hospital system participated in the RCR, only encounters within the health system’s affiliated hospitals were available to categorize deceased participants’ last known encounter type. The last recorded encounter type for deceased patients was either an inpatient (46.3%) or outpatient (46.3%) visit. On average, the duration from the previous encounter to the date of death was 37.8 days. Duration’s distribution was negatively skewed, with extreme outliers resulting in a very large standard deviation of 88.1 days.

**TABLE 5:** Deaths across all trial participants.

Number of enrolled trials^a^	
One study	59 (88.1)
More than one study	8 (11.9)
Last encounter type^a^ ^,^ ^b^	
Outpatient	31 (46.3)
Inpatient	31 (46.3)
Research	4 (6.0)
Emergency Department	1 (1.5)
Days to death from last encounter^c^ ^,^ ^d^	37.8 (88.1)

^a^ Frequency [Relative Frequency (%)].

^b^ Only encounters within the health system’s affiliated hospitals were available to categorize the last known encounter type for deceased participants.

^c^ Mean (Standard Deviation).

^d^ Calculated as the difference between the last known encounter date and date of death.

The binary logistic regression model identified eight variables associated with trial death at last known follow-up (Table 6). Age at initial trial screening, residence region, and residence rurality were significantly associated with trial death. Specifically, older age, those residing in Northeastern Montana, and those living in metropolitan RUCA codes had significantly higher odds of death. Furthermore, participants enrolled from 1999 to 2020 and in more than one trial had significantly higher odds of death. Participants undergoing cancer treatment and those in Phase 1 or 2 trials had exceptionally high odds of death. While domestic trial sponsors had significantly higher odds of death, the type of trial sponsor (e.g., academic institute) was of no predictive value. Marital status, gender, race, insurance status, insurance type, and study status were each eliminated as highly non-significant factors during the backward selection methodology to fit the logistical regression mode.

**TABLE 6:** Logistic regression model for trial death.

Factor^a^	Odds ratios for predictors			Coefficients^c^	
Level A	Level B^b^	OR	Or 95% CI	β	*p*-value
Age at screening	-	1.09	(1.06, 1.12)	0.09	<0.001**
Study phases Observational	Pilot	0.47	(0.02, 4.65)	−0.75	0.557
	I	209.1	(32.6, 1,684.5)	5.34	<0.001**
	II	3.93	(1.30, 11.51)	1.37	0.013*
	III	2.42	(0.80, 7.06)	0.88	0.109
	IV	0.63	(0.19, 2.11)	−0.46	0.447
Montana region Eastern	Central & Western	2.26	(0.77, 6.12)	0.81	0.120
	Northeastern	3.55	(1.18, 11.05)	1.27	0.025*
	Outside MT	0.46	(0.11, 1.51)	−0.79	0.231
Disease Site Cancer, Breast & Gynecological	Diabetes	0.13	(0.03, 0.50)	−2.04	0.003**
	Other	2.91	(0.95, 9.62)	1.07	0.069
Study Type Domestic	International	0.13	(0.05, 0.33)	−2.08	<0.001**
RUCA Metropolitan	Non-metropolitan Area	0.42	(0.17, 0.95)	−0.86	0.045*
Enrolled Year 2020 and prior	2021–2022	0.22	(0.09, 0.48)	−1.52	<0.001**
Trials enrolled One study	More than one study	2.31	(1.11, 4.74)	0.84	0.023*
Sponsorship Biotechnology/Device	Academic institute/Health Org	0.29	(0.07, 1.07)	−1.25	0.073
	Consortium/Network/Foundation	0.48	(0.13, 1.73)	−0.74	0.258
	Pharmaceutical/Biopharmaceutical	2.01	(0.61, 6.35)	0.70	0.240

^a^ Only statistically significant factors/levels and near/close to be significant factors and their levels were listed.

^b^ Odd ratios for level A to level B.

^c^ Coefficients of level A: * *p*-value is less than 0.05; ** *p*-value is less than 0.01.

^d^ Median number of diagnoses was 10, median of the number of procedures was 2, and median length of stay was 5 days.

## 4 Discussion

This retrospective chart review examined patients’ medical records enrolled in a clinical trial between 1999 and 2022 at a large healthcare organization in the rural Northwest. The final dataset of 833 records included 753 individual participants, with 75 patients participating in more than one trial during the study period. Numerous other studies have been conducted on this topic; however, none were found relevant to the rural frontier population of our service area.

Research participants were, on average, 57.5 years of age at their first clinical trial screening, and 3.6% of participants enrolled in clinical trials were 80 years or older, a larger number than expected. These findings do not align with the current literature, where clinical trial participants are, on average, 55 years of age or younger (NIH, 2021). However, the findings do align with a recent report from the US Census Bureau stating that older adults comprise 17.5% of the rural population, in contrast to urban centers at 13.8% (Smith and Trevelyan, 2019).

Most clinical trial participants in this sample were women (62.2%), which is a higher number than reported in the literature. A recent study found that low recruitment of women remains an issue in industry-sponsored early-phase trials, with females accounting for 29%–34% of participants (Cottinham and Fisher, 2022). However, the higher-than-average participation of women in clinical trials in our region correlates with clinical service delivery as breast and gynecological cancers are two of the five top-treated cancers at our healthcare facility. In this sample, 172 (20.6%) of patients participated in breast/gynecological clinical trials, just behind 284 (34.1%) participants enrolled in diabetes clinical trials. Most trials in our sample were observational; however, interventional clinical trials continue to increase and diversify across the region. Clinical trials in our area primarily focus on diabetes, breast or gynecological cancer, and other forms of cancer. Due to the region’s lack of availability of specialty services, very few Biobank/Repository and lung-related trials were conducted.

While most participants were white, 4.5% of participants in this study were American Indian/Alaska Native, a percentage much higher than the national average of 1% (Mainous et al., 2023). This percentage is comparable to our service area metrics which show around 5% of our population is American Indian/Alaskan Native. This higher number may be attributed to the large service area of our region, including 7 American Indian reservations and 12 American Indian tribes (OPI, 2015). As the largest healthcare system in the rural Northwest, clinical service reaches a diverse population of varying ethnical and racial backgrounds across Montana, Wyoming, Idaho, and the Dakotas.

In analyzing our dataset, we found that 46.8% had commercial insurance and 49.4% had government-issued insurance. We also identified that over 33% had two or more insurances which would align with the aging population served in clinical trials. Even though the majority of services may be covered under the standard of care or via clinical trial benefits, patients may still be expected to pay copays and coinsurance, which may deter clinical trial participation (Unger et al., 2021; Brøgger-Mikkelsen et al., 2022).

While most participants were from metropolitan areas (i.e., RUCA 1 and 2), nearly 22% of participants lived in rural areas, with 10% living in frontier areas. Most participants (71.9%) lived closest to the large healthcare center. This finding aligns with current literature where the distribution of clinical trial participants is largely urban versus rural (Seidler et al., 2014; de Jong et al., 2022). From a practical standpoint of conducting clinical research, it is understandable to see a greater number of participants from urban areas with improved access to healthcare services. With only seven NIH top research centers in rural areas compared to the 49 in urban centers, the awareness and socialization of populations to research remains different between geographic regions (Brogger-Mikkelsen et al., 2022). Expanding clinical research to rural areas and exploring opportunities for innovative trial methodologies and designs, such as decentralized trials, may improve the heterogeneity of the sample population and the generalizability of the research findings.

Deceased participants were, on average, 10 years older than surviving participants, with half of the deaths occurring in those aged 70 years and older and among participants enrolled in one clinical trial. Most notably, death was highest among metropolitan tracts (67.5%) compared to non-metro tracts (32.5%). Specifically, participants who were older, living in the Northeastern Montana region, and residing in a metropolitan area had significantly higher mortality odds. Participants undergoing cancer treatment and those in Phase 1 or 2 trials had exceptionally high odds of death. When comparing deaths among Montana residents to the US average (Table 7), Montana participants (≥65 years old) in cancer clinical trials have lower death rates (3.2%) than the national mortality average of 4.7% (Census 2020). Future research is needed to further explore mortality differences among urban and rural clinical trial research participants using epidemiological surveillance.

**TABLE 7:** Difference for RCR study’s relative death frequency and current Mortality US rates (Census 2020).

Age interval (years)	Trial death frequency (all causes) (%)	US mortality rate (all causes) (%)	Montana mortality rate (all causes)	US mortality rate (cancer) (%)	Montana mortality rate (cancer) (%)	Montana mortality rate (heart diseases) (%)
18–44	7.8	0.5^a^	1.5%^a^	0.07^a^	0.04^a^	0.03^a^
45–64	31.2	1.5	3.3%	0.7	0.3	0.3
≥65	61.0	22.3	30.4	4.7	3.2	5.2

^a^ Includes mortality rate statistics for 15–18 age interval.

These findings suggest a need for targeted interventions to improve access to and education about clinical trials in rural and frontier areas. Additional work needs to be conducted to continue to gain trust in the rural communities and find ways to help with socioeconomics considerations of these patients. As the populations of our rural communities continue to age, an increased need for research services closer to patients’ homes will become vitally important.

## 5 Conclusion

This RCR described the adult clinical trial participation landscape across a large healthcare organization in the rural Northwest between 1999 and 2022. While the majority of trials were observational in nature, there were significant portions of the enterprise portfolio that centered on interventional research with industry partners, particularly surrounding diabetes and cancer. The older age of the sample population aligns with the general population of Montana and demonstrates a willingness of older adults to engage in research. While a higher likelihood of death was associated with early-phase and cancer-related research, this aligns with national assumptions with safety/efficacy studies with accelerated, varied disease processes such as cancer.

As decentralized trial design and remote or virtual elements of traditional trials become normative, representation of rural and frontier populations is not only possible but imperative to support the generalizability of trial data encouraged by the FDA. The increase in participation during and after the pandemic demonstrates the successful engagement of non-urban communities, which is critical to future awareness initiatives with novel therapies and technologies (e.g., remote patient monitoring). Retrospective chart reviews, such as the one conducted, support organizational readiness for expanded and varying the types of clinical trials within the enterprise portfolio, given the projection potential derived from historical data. Furthermore, a critical analysis of safety management policies and procedures is possible, given population-based mortality data and aggregated participant demographic information. Healthcare systems serving rural and frontier populations, empowered by historical data analysis, can lead change in expanding opportunity equity and conducting culturally congruent clinical trials in the communities served.

## 6 Limitations

Logistic regression analysis is a statistical technique to evaluate the relationship between predictor variables and a dichotomous outcome, which may have been impacted by concurrent effects of several predictor factors not controllable during the current study. While care was taken to capture all clinical trial participants within the review period, there is a possibility of missing potential eligible cases due to transcription error at the time of record creation or lack of consent form upload into the EHR. Additionally, survival analysis is the best practice to analyze clinical trial treatment efficacy. This study’s RCR was unable to capture critical temporal effects (e.g., longitudinal follow-up data across many times points) except trial status at last known follow-up. This study was also unable to prospectively enroll participants in trials or treatment types. Future studies based on the results of this exploratory analysis of an extremely heterogenous mixtures of trials will utilize survival analysis to understand trial enrollment, barriers, and treatment efficacy for rural participants.

Due to low sample sizes, IRB restrictions and confidentiality, this RCR was unable to complete and/or report detailed findings related to race, ethnicity, and rurality. Future prospective studies will capture the necessary consent and data to better understand clinical trial barriers particularly among BIPOC and frontier residents.

This RCR did not include children and youth under the age of 18, which limited the interpretation to characteristics of clinical trial participation to adults. However, pediatric clinical trials are conducted at the organization, and future research will include a sub-set examination of children’s enrollment in research.

## Data Availability

The data analyzed in this study is subject to the following licenses/restrictions: Deidentified data available upon request. Requests to access these datasets should be directed to JN, jbesel@billingsclinic.org.
